# Antibacterial activity of recently approved antibiotics against methicillin-resistant *Staphylococcus aureus* (MRSA) strains: A systematic review and meta-analysis

**DOI:** 10.1186/s12941-022-00529-z

**Published:** 2022-08-17

**Authors:** Fei Liu, Sajad Rajabi, Chunhua Shi, Ghazale Afifirad, Nazanin Omidi, Ebrahim Kouhsari, Saeed Khoshnood, Khalil Azizian

**Affiliations:** 1 Department of Biomedical Engineering, Changzhi Medical College, Changzhi, 046013 Shanxi China; 2 International Medical Campus, Iran University of Medical Sciences, Tehran, Iran; 3 Department of Microbiology, School of Medicine, Tehran University of Medical Sciences, Tehran, Iran; 4 Clinical Microbiology Research Center, Ilam University of Medical Sciences, Ilam, Iran; 5 Laboratory Sciences Research Center, Golestan University of Medical Sciences, Gorgan, Iran; 6 Department of Laboratory Sciences, Faculty of Paramedicine, Golestan University of Medical Sciences, Gorgan, Iran; 7 Department of Microbiology, Faculty of Medicine, Kurdistan University of Medical Sciences, Sanandaj, Iran

**Keywords:** MRSA, Antibacterial activity, Tedizolid, Telavancin, Dalbavancin, Oritavancin, Lipoglycopeptide

## Abstract

**Background:**

Methicillin-resistant *Staphylococcus aureus* (MRSA) infections are considered an important public health problem, and treatment options are limited. Accordingly, in this meta-analysis, we analyzed published studies to survey in vitro activity of recently approved antibiotics against MRSA isolates.

**Methods:**

We searched electronic databases; PubMed, Scopus, and Web of Science to identify relevant studies (until November 30, 2020) that have focused on the in vitro activity of telavancin, dalbavancin, oritavancin, and tedizolid against MRSA isolates. Statistical analyses were conducted using STATA software (version 14.0).

**Results:**

Thirty-eight studies were included in this meta-analysis. Overall in vitro activity of tedizolid on 12,204 MRSA isolates was 0.250 and 0.5 µg/mL for MIC_50_ and MIC_90_, (minimum inhibitory concentration at which 50% and 90% of isolates were inhibited, respectively), respectively. The overall antibacterial activity of dalbavancin on 28539 MRSA isolates was 0.060 and 0.120 µg/mL for MIC_50_ and MIC_90_, respectively. The overall antibacterial activity of oritavancin on 420 MRSA isolates was 0.045 and 0.120 µg/mL for MIC_50_ and MIC_90_, respectively. The overall antibacterial activity of telavancin on 7353 MRSA isolates was 0.032 and 0.060 µg/mL for MIC_50_ and MIC_90_, respectively. The pooled prevalence of tedizolid, telavancin, and dalbavancin susceptibility was 100% (95% CI: 100–100).

**Conclusion:**

Telavancin, dalbavancin, oritavancin, and tedizolid had potent in vitro activity against MRSA isolates. The low MICs and high susceptibility rates of these antibiotics recommend a hopeful direction to introduce useful antibiotics in treating MRSA infections in the future.

**Supplementary Information:**

The online version contains supplementary material available at 10.1186/s12941-022-00529-z.

## Introduction

*Staphylococcus aureus* (*S. aureus)* is a prominent cause of hospital-acquired and community-acquired infections ranging from superficial skin and soft tissue infections to endocarditis [1, 2].

For two reasons, A) methicillin-resistant *Staphylococcus aureus* (MRSA) is a well-recognized public health problem worldwide [3], and B) Antibiotic-resistance pattern of MRSA. Currently, World Health Organization (WHO) considers *S. aureus,* especially MRSA, as one of the fundamental clinical challenges throughout the world. [4]. There are limited therapeutic options for the treatment of MRSA infections. Vancomycin is introduced as a drug of choice for treating serious infections due to MRSA. However, overuse of vancomycin leads to the emergence of non-susceptible strain [5–7]. For example, vancomycin-resistance *S. aureus* (VRSA) strains have been reported from many countries, including the USA, India, Iran, and Pakistan [5–7].

Furthermore, linezolid and clindamycin are other favorable antibiotics against MRSA infections [8]. Despite different mechanisms of action, the emergence of resistant strains to these antibiotics is rising [8–12]. Increased antibiotic resistance in MRSA isolates is one of this century's most globally significant problems [4]. Several new agents such as telavancin, dalbavancin, oritavancin, and tedizolid have recently been licensed for the treatment of infections caused by MRSA.

Following the emergence of strains with reduced susceptibility to vancomycin (first generation of glycopeptide), the second generation of semisynthetic lipoglycopeptides has been developed as alternatives for treating MRSA infections. Telavancin, dalbavancin, and oritavancin have been introduced as critical lipoglycopeptide antibiotics recently approved by the Food and Drug Administration (FDA). Telavancin was the first approved lipoglycopeptide by the FDA in 2009 [13]. Furthermore, dalbavancin and oritavancin were first approved by the FDA in 2014 [14, 15]. Lipoglycopeptides are semisynthetic derivatives characterized by adding a lipophilic side chain, which prolongs their half-lives and increases their activities against Gram-positive cocci [16]. Lipoglycopeptides inhibit cell wall synthesis by binding to C-terminal D-alanyl-D-alanine (D-Ala-D-Ala) of cell wall precursor units [17, 18]. The N-alkyl-p-chlorophenylbenzyl substituent in oritavancin confers significantly enhanced activity against vancomycin-intermediate and-resistant staphylococci [17]. In addition, lipoglycopeptides can interfere with cellular membrane functions [17, 19].

Linezolid, the first oxazolidinone antibacterial agent, was approved in the United States in early 2000. The following approved oxazolidinone was tedizolid. Tedizolid is a second-generation oxazolidinone class approved in 2014 by the FDA. This antibiotic is a bacteriostatic compound against gram-positive bacteria [20]. Similar to linezolid, the mechanical action of tedizolid is inhibiting protein synthesis by binding to the 23S ribosomal RNA of the 50S subunit [21]. Tedizolid is an oxazolidinone but differs from other oxazolidinones by possessing a modified side chain at the C-5 position of the oxazolidinone nucleus that improves potency through additional binding site interactions [22]. Not many in-depth studies are available that directly compare the susceptibilities of telavancin, dalbavancin, oritavancin, and tedizolid to different MRSA strains. Therefore, this systematic meta-analysis was conducted to survey in vitro activity of recently approved antibiotics against MRSA isolates by analyzing the related published studies.

## Methods

### Guidelines

This review is reported according to the Preferred Reporting Items for Systematic Reviews and Meta-Analyses guidelines (PRISMA) [23].

### Search strategy

**Fig. 1:**
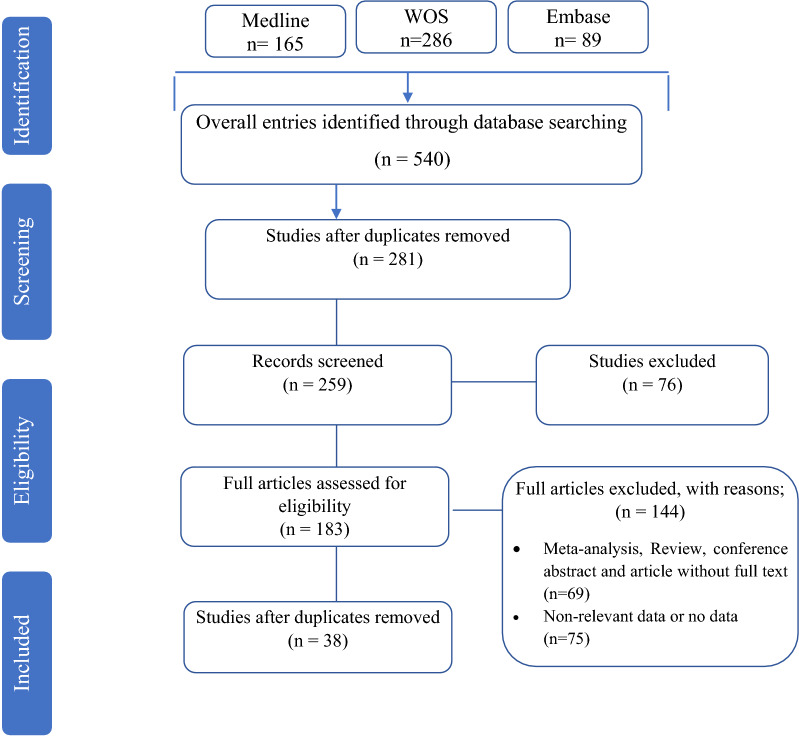
PRISMA flow chart of the article selection procedure

A systematic search was conducted to evaluate the antibacterial activity of recently approved antibiotics against MRSA strains. The electronic databases: Medline, Embase, and Web of Science were searched to identify relevant articles until November 30, 2020. The search strategy was based on keywords derived from our research questions. The keywords used in the search were: "tedizolid", "dalbavancin", "oritavancin", "telavancin", "delafloxacin", "Methicillin-Resistant *Staphylococcus aureus*", and "minimum inhibitory concentration". The Boolean operators were used to combine all descriptors. The search strategy was adapted to the features of each database. If possible, we searched for synonyms or used the search option for similar terms before every keyword. No limitation was applied during the searching procedure of databases, but the inclusion of the study in our full analysis required at least the English abstract to be available. The records found through database searching were merged, and the duplicates were removed using EndNote X7 (Thomson Reuters, New York, NY, USA). Reference lists of all eligible articles were also reviewed to find any additional potentially relevant studies. The flow chart of the selected articles is shown in Fig. 1.

### Eligibility criteria

Identified studies that were consistent with the criteria included original articles published in English concerning the antibacterial activity of recently approved antibiotics against MRSA strains. After screening, duplicate studies, non-original articles (reviews, short communications, case studies, abstracts without full text, and book chapters), and studies that lack information regarding the minimum inhibitory concentration (MIC) were excluded.

One reviewer performed the searches; then, initial screening was done by two independent reviewers for potentially relevant records matching the inclusion/exclusion criteria based on title and abstracts. Full articles were obtained from these records and were assessed for relevance by two independent reviewers. Any discrepancies with the third reviewer were resolved by consulting. Whereas the initial study was not available, requests were made to the authors.

### Data extraction and quality assessment

Two reviewers coded and extracted the data independently. This process was also overseen by the third author again. All studies were consistent with the following inclusion criteria: (1) antibacterial activity was determined using one of the standard methods, including broth microdilution, agar dilution, and epsilometer (E)-test, (2) MIC_50_ and MIC_90_ (minimum inhibitory concentration at which 50% and 90% of isolates were inhibited, respectively) and their ranges were available, also (3) original studies that were performed on clinically derived isolates. Meanwhile, exclusion criteria were (1) studies that have not reported the MIC or have not used the standard susceptibility testing methods, (2) studies with a sample size < 10 isolates, and (3) studies performed on samples with animals or environment origin. Neither reviews nor systematic review articles, case reports, and articles available only in the abstract that lacks necessary information were included. Moreover, the quality of included studies was critically appraised using the Newcastle–Ottawa Scale [24]. The pre-defined review protocol was registered at the PROSPERO international prospective register of systematic reviews (http://www.crd.york.ac.uk/PROSPERO, registration number CRD11111).

### Statistical analysis

The meta-analysis was performed by computing the pooled using a random-effects model with Stata/SE software, v.17 (StataCorp, College Station, TX) on studies presenting raw data on antibacterial activity of tedizolid, dalbavancin, oritavancin, telavancin, and delafloxacin against MRSA strains. The inconsistency across studies was examined by the forest plot as well as the I^2^ statistic. Values of I^2^ (25%, 50%, 75%) were interpreted as the presence of low, medium, or high heterogeneity, respectively. So, the DerSimonian and Laird random effects models were used [25]. Publication bias was assessed using Egger's test. All statistical interpretations were reported on a 95% confidence interval (CI) basis.

### Study outcomes

The primary outcome of interest was the pooled prevalence susceptibility of tedizolid, dalbavancin, and telavancin against MRSA isolates. The secondary outcomes of interest were the MIC_50_ and MIC_90_ of tedizolid, dalbavancin, and telavancin against MRSA isolates.

## Results

### Systematic literature search

A total of 540 records were identified in the initial search. Among these, 357 articles were excluded after an initial screening of the title and abstract due to their irrelevance and duplication. The full texts of the remaining 183 articles were reviewed (Fig. 1). Out of 183 articles, 145 were excluded for the following reasons: meta-analysis, review, conference abstract, and article without full text (n = 70), non-relevant data, or no MIC data (n = 75). Finally, the detailed characteristics of 38 included studies in this meta-analysis are indicated in Table 1.

**Table 1:** The details included studies

First author	Study period	Publication year	Quality score	Continents/countries	Sample source	No. MRSA isolates	Type of antibiotics	MIC50/MIC90 (µg/ml)	MIC range (µg/ml)	Susceptibility rate (%)	References
Gulseren Aktas	2005 and 2007	2010	7	Turkey	clinical isolates	237	Dalbavancin	≤ 0.008 / 0.25	≤ 0.008–2	99.6	[44]
Gulseren Aktas	2014 and 2015	2016	7	Turkey	clinical isolates	30	Dalbavancin	0.12 / 0.12	0.03–0.12	100	[45]
Maya Azrad	2015 and 2017	2019	7	Israel	Blood sample, Wounds	275	Tedizolid	0.25 / 0.3	0.19–0.5	100	[46]
						275	Dalbavancin	MIC50: 0.047 / MIC90:0.055 (wound)/0.06 (Blood sample)	0.023–0.19	99.64	[46]
Diane M. Citron	No data	2014	6	USA	osteomyelitis	15	Dalbavancin	0.06 /0.06	≤ 0.03–0.12	No data	[47]
G. Ralph Corey	2011 to 2013	2016	7	USA	blood culture and ABSSSI	405	Oritavancin	0.03 / 0.12	0.002–0.25	99.1	[48]
Ko-Hung Chen	2013 to 2014	2015	7	Taiwan	ABSSSI and pneumonia	ABSSSI (50) and pneumonia (50)	Tedizolid	ABSSSI (0.25/0.25) and pneumonia (0.5/0.5)	ABSSSI (0.25/0.5) and pneumonia (0.25/0.5)	ABSSSI (100) and pneumonia (100)	[21]
Yong Pil Chong	2004 to 2009	2012		Korea	blood cultures	569	Dalbavancin	0.25 / 0.25	0.06–0.25	98.8	[49]
Aneta Guzek	2012 to 2014	2018	7	Poland	clinical isolates	124	Dalbavancin	0.094/0.125	0.032–0.125	100	[50]
Vanthida Huang	No data	2010	7	USA	220 clinical isolates	CA-MRSA (110), MDR HA-MRSA (n = 110)	Dalbavancin	CA-MRSA: 0.0625/0.125, MDR HA-MRSA: 0.125/0.125	CA-MRSA (0.0625–0.125), MDR HA-MRSA (0.03125–0.25)	No data	[51]
Ronald N. Jones	2011–2014	2017	7	North American, Latin American, European, and Asia–Pacific Nations	bone and joint	229	Telavancin	0.03 / 0.06	≤ 0.015–0.06	100	[52]
James A. Karlowsky	2014 to 2016	2017	7	Asia/Pacific region (Australia [n = 2], China [n = 16], New Zealand [n = 2], Philippines [n = 2], Taiwan [n = 2]), the Latin America region (Argentina [n = 2], Brazil [n = 10], Chile [n = 2], Colombia [n = 2], Mexico [n = 6]), Russia (n = 7), and Saudi Arabia (n = 1)	ABSSSI, blood samples, respiratory infections	1839	Tedizolid	0.25 / 0.5	0.03–0.5	100	[53]
Yangsoon Lee	2011 to 2014	2015	7	Korea	SSSIs, HAP	skin and skin structure infections (90), hospital-acquired pneumonia (61)	Tedizolid	skin and skin structure infections (0.5 / 0.5), hospital-acquired pneumonia (0.25/ 0.5)	skin and skin structure infections (0.125–0.5), hospital-acquired pneumonia (0.125–0.5)	100	[54]
María Carmen López-Díaz	2012 to 2014	2017	7	Spain	clinical isolates	55	Dalbavancin	0.125 / 0.125	0.06–0.125	100	[55]
Johanna Marcela Vanegas Múnera	2008 to 2010	2017	7	Colombia	clinical isolates	150	Tedizolid	0.38 / 0.5	≤ 0.19–0.75	100	[56]
Rodrigo E. Mendes	2011 to 2013	2015	7	USA	clinical isolates	4651	Telavancin	0.03 / 0.06	≤ 0.015–0.12	100	[57]
Sandra P. McCurdy	2002–2012	2015	7	USA, Europa, Russian and Israeli	clinical isolates	26975	Dalbavancin	0.06 / 0.06	< 0.008–0.5	99.6	[58]
R. E. Mendes	2011–2014	2017	7	North America (2150 isolates), Europe (1283), Latin America (473), and Asia–Pacific (APAC; 285) regions	blood samples	1490	Telavancin	0.03 / 0.06	≤ 0.015–0.12	100	[59]
Jessica Baleiro Okado	2011- 2012	2018	7	Brazil	clinical isolates	27	Tedizolid	0.25 / 0.25	0.125–0.5	100	[60]
Michael A. Pfaller	2014–2015	2019	7	USA	SSSIs, bacteremia, pneumonia, intra-abdominal infections, urinary tract infections	1732	Tedizolid	0.12 / 0.12	0.03–0.25	100	[61]
Philippe Prokocimer	2008–2009	2012	7	USA	clinical isolates	124	Tedizolid	0.25 / 0.25	0.12–0.5	100	[62]
Kenneth VI Rolston	2012–2013	2014	7	USA	clinical isolates	50	Telavancin	0.25 / 0.25	0.064–0.38	No data	[63]
Laser Sanal	2013–2016	2018	7	Turkey	blood and tracheal aspirate	50	Telavancin	0.032 / 0.064	0.016—0.125	100	[64]
Suzannah M. Schmidt-Malan	1996–2014	2016	7	USA	clinical isolates	35	Tedizolid	0.5 / 0.5	0.25–0.5	100	[65]
Wael Shams	1991–2006	2010	7	USA	bloodstream, respiratory tract and wound	168	Telavancin	0.25 / 0.50	0.08–1.00	No data	[66]
Debora Sweeney		2017	7	USA	clinical isolates	15	Oritavancin	0.06 / 0.12	0.03–0.12	100	[67]
							Dalbavancin	0.03 / 0.06	0.03–0.06	100	[67]
							Telavancin	0.06 / 0.06	0.06–0.12	100	[67]
							Tedizolid	0.25 / 0.5	0.25–0.5	100	[67]
Jennifer I. Smart	No data (2008)	2016	7	USA	SSSIs	700	Telavancin	0.06/0.06	0.03–0.12	100	[68]
Kenneth S. Thomson	No data	2013	7	USA	clinical isolates	111	Tedizolid	0.5 / 0.5	0.12–0.5	100	[69]
Floriana Campanile	2005–2007	2010	7	Italia	bloodstream, pneumonia, and SSSIs	24	Dalbavancin	0.06 / 0.12	0.03–0.12	100	[70]
D. J. Biedenbach	2013–2014	2016	7	Argentina, Brazil, Chile, Mexico, Australia and New Zealand, china	clinical isolates	Argentina, Brazil, Chile, and Mexico (318), Australia and New Zealand (51) china (425)	Tedizolid	Argentina, Brazil, Chile,and Mexico (0.5/0.5), Australia and New Zealand (0.25 / 0.5), china (0.25 / 0.5)	0.12–0.5	100	[39]
Carmen Betriu	2004–2008	2010	7	Spain	Blood samples	247	Tedizolid	0.25/0.5	0.125–0.5	100	[40]
Mekki Bensaci	2009–2013	2017	7	USA	clinical isolates	3234	Tedizolid	0.25/0.5	≤ 0.015 to 2	99.6	[71]
Hongbin Chen	2009–2013	2014	7	China	SSSIs, lower respiratory tract infections	100	Tedizolid	0.25 / 0.25	0.064–1	No data	[72]
Steven D. Brown	No data	2010	7	USA	clinical isolates	129	Tedizolid	0.5 /1	0.12–16	No data	[73]
Jong Hwa Yum	2002–2004	2010	7	South Korea	clinical isolates	30	Torezolid	0.5/0.5	0.5	100	[74]
Daniel F. Sahm	2011–2012	2015	7	USA, Europa	clinical isolates	1770	Tedizolid	0.25/0.5	0.015–4	99.7	[75]
Shuguang Li	2014	2016	7	China	clinical isolates	632	Tedizolid	0.25 / 0.25	0.064–0.5	100	[76]
Marina Peñuelas	No data	2016	7	Spain	clinical isolates	18	Tedizolid	0.25/0.5	0.125–0.5	100	[77]
Michael A. Pfaller	2014	2016	7	Asia–Pacific, Eastern Europe, and Latin American Countries	clinical isolates	701	Tedizolid	0.12/0.12	0.03–0.25	100	[78]

*ABSSSI* acute bacterial skin and skin structure infections, *CA-MRSA* community-associated MRSA infections, *HA-MRSA* healthcare-acquired methicillin-resistant *Staphylococcus aureus*, *HAP* hospital-acquired pneumonia

### Characteristics of included studies

All included studies had a cross-sectional design. All included studies in this meta-analysis were high-quality (Additional file 2: Table) [24]. However, most reports have been from America (n = 19), Asia (n = 8), Europe (n = 8), and multiple continents (n = 7). In the current study, to determine the effective concentration of tedizolid, dalbavancin, oritavancin, and telavancin against MRSA isolates, the mode of MIC_50_, MIC_90_, and MIC ranges was estimated (Table 2). To analyze the trends for changes in the tedizolid, dalbavancin, oritavancin, and telavancin susceptibility in recent years, we performed a subgroup analysis for two periods (2010–2015 and 2016–2020) (Tables 3, 5, Additional file 1: Figure). No significant difference was observed in the pooled prevalence of tedizolid, dalbavancin, oritavancin, and telavancin susceptibilities against MRSA isolate for two periods (2010–2015 and 2016–2020) (Tables 3, 4, 5).

**Table 2:** Antibacterial activity of mentioned antibiotics against MRSA isolates

Variable	MIC_50_	MIC_90_	MIC range	
			Min.	Max.
Dalbavancin				
Mode	0.060	0.120	0.030	0.220
Min.	0.008	0.060	0.023	0.120
Max.	0.250	0.250	0.250	2.000
Oritavancin				
Mode	0.045	0.120	0.033	0.625
Min.	0.030	0.120	0.002	0.250
Max.	0.060	0.120	0.064	1.000
Tedizolid				
Mode	0.250	0.500	0.030	0.500
Min.	0.120	0.120	0.015	0.060
Max.	0.500	1.000	0.250	4.000
Telavancin				
Mode	0.032	0.060	0.064	0.500
Min.	0.030	0.060	0.030	0.120
Max.	0.250	0.500	0.250	16.000

**Table 3:** Antibacterial activity of dalbavancin against MRSA isolates based on year groups

Dalbavancin		MIC_50_	MIC_90_	MIC_50_/_90_	Susceptibility rate (%)
2010–2015	Median	0.06	0.1225	1	100
	Min.	0.008	0.06	1	98.8
	Max.	0.25	0.25	31.25	100
2016–2020	Median	0.094	0.12	1.276	100
	Min.	0.03	0.06	1	99.64
	Max.	0.125	0.125	2	100

**Table 4:** Antibacterial activity of telavancin against MRSA isolates based on year groups

Telavancin		MIC_50_	MIC_90_	MIC_90/50_	Susceptibility rate (%)
2010–2015	Median	0.25	0.25	2	100
	Min.	0.03	0.06	2	100
	Max.	0.25	0.5	1	100
2016–2020	Median	0.032	0.06	2	100
	Min.	0.03	0.06	1	100
	Max.	0.06	0.064	2	100

**Table 5:** Antibacterial activity of tedizolid against MRSA isolates based on year groups

Tedizolid		MIC_50_	MIC_90_	MIC_50_/_90_	Susceptibility rate (%)
2010–2015	Median	0.25	0.5	1	100
	Min.	0.25	0.25	1	99.7
	Max.	0.5	1	2	100
2016–2020	Median	0.25	0.5	1.2	100
	Min.	0.12	0.12	1	99.6
	Max.	0.5	0.5	2	100

### Antibacterial activity of tedizolid

The prevalence of tedizolid susceptibility is available in 21 studies. The overall antibacterial activity of tedizolid in 12,204 MRSA isolates was at 0.250 and 0.500 µg/mL for MIC_50_ and MIC_90_, respectively. Out of 21 studies, the pooled prevalence of tedizolid susceptibility was 100% (95% CI: 100–100) (Table 6). There was no substantial heterogeneity among the 21 studies (*p* = 0.99; I^2^ = 0%).

**Table 6:** The pooled prevalence susceptibility of tedizolid, oritavancin, dalbavancin, and telavancin against MRSA isolates

Antibiotics	Number of studies	Number of MRSA Isolates	Proportion (95% CI)	chi^2^	Heterogeneity P	I^2^	p
Dalbavancin	11	28539	1.00, (1.00,1.00)	8.22	0.61	0.00%	0.00
Oritavancin	2	420	1.00, (1.00,1.00)				0.00
Tedizolid	21	12204	1.00, (1.00,1.00)	7.57	0.99	0.00%	0.00
Telavancin	8	7353	1.00, (1.00,1.00)	3.26	0.86	0.00%	0.00

### Antibacterial activity of dalbavancin

The prevalence of dalbavancin susceptibility is available in 11 studies. The overall antibacterial activity of tedizolid was 0.060, and 0.120 µg/mL for MIC_50_ and MIC_90_ in 28539 MRSA isolates, respectively. Out of 11 studies, the pooled prevalence of dalbavancin susceptibility was 100% (95% CI: 100–100) (Table 6). There was no substantial heterogeneity among the 11 studies (*p* = 0.61; I^2^ = 0%).

### Antibacterial activity of oritavancin

The prevalence of oritavancin susceptibility was available in 2 studies. The overall antibacterial activity of oritavancin was 0.045, and 0.120 µg/mL for MIC_50_ and MIC_90_ in 420 MRSA isolates, respectively.

### Antibacterial activity of telavancin

The prevalence of telavancin susceptibility was available in 8 studies. The overall antibacterial activity of telavancin was 0.032, and 0.060 µg/mL for MIC_50_ and MIC_90_ in 7353 MRSA isolates, respectively. From 8 studies, the pooled prevalence of telavancin susceptibility was 100% (95% CI: 100–100) (Table 6). There was no substantial heterogeneity among the eight studies (*p* = 0.86; I^2^ = 0%).

## Discussion

MRSA is considered one of the most critical human health problems worldwide [26]. Empirical therapies by vancomycin and linezolid were reliable options for treating MRSA infections [27]. However, reports on decreasing susceptibility to vancomycin and linezolid are worrying [28]. It is critical to introduce and characterize new effective and safe antibiotics to prevent and control the infections related to MRSA strains [29]. The findings from a systematic review demonstrated that the prevalence of VRSA increased in recent years around the world [30]. It also was shown that different continents and countries are struggling with VRSA strains [30].

Compared with the classic glycopeptides, our meta-analysis shows a higher antibacterial activity of a new class of lipoglycopeptides (telavancin and dalbavancin susceptibilities were 100%). Moreover, the estimated MIC values of three lipoglycopeptides (MIC_50_/MIC_90_, 0.060/0.120 µg/mL for dalbavancin, MIC_50_/MIC_90_, 0.032/0.060 µg/mL for telavancin, MIC_50_/MIC_90_, 0.045/0.120 µg/mL for oritavancin) against MRSA strains are much lower than the MIC value of vancomycin for MRSA in the literature [31]. Moreover, against both vancomycin-resistant *Enterococcus* (VRE) and vancomycin-susceptible *Enterococcus* (VSE), the MIC value of lipoglycopeptides is much lower than the MIC value of vancomycin [16].

MIC_50_/_90_ values of dalbavancin (0.06/0.12 µg/mL) are very similar to another systematic review published by Sadr in 2017 [32]. Moreover, compared to vancomycin, previous studies indicated that dalbavancin showed potent activity against biofilm-forming bacteria [33, 34]. However, a network meta-analysis showed no significant differences between dalbavancin and vancomycin in treating acute bacterial skin and soft-tissue infections (SSTIs) [35]. Dalbavancin susceptibility was more than 99% in the published systematic review in 2017, as our results [32].

In our study, the MIC_50_ value of oritavancin against MRSA strains is similar to a systematic review by Mendes et al. in 2015 [36]. Solo clinical trials show that oritavancin is more effective than vancomycin against MRSA infections [37]. Mendes et al. [36] evaluated the activity in vitro of oritavancin and comparators against Gram-positive pathogens causing SSTIs in European and US hospitals. They showed that oritavancin susceptibility in Gram-positive clinical isolates from the United States and Europe were 98.4% and 98.9%, respectively [36]. However, our meta-analysis studied worldwide data, and oritavancin susceptibility was 100%.

A previous systematic review and meta-analysis published in 2019 reported that the MIC_50_ and MIC_90_ of tedizolid were 0.250 and 0.500 µg/mL, respectively [38]. These MIC values are lower than the MIC values of vancomycin against MRSA strains [39, 40]. It was also shown that the MIC of tedizolid is much lower than the MIC of vancomycin against VISA strains [41]. In addition, tedizolid demonstrated greater in vitro potency than linezolid against MRSA strains, but further research is required for a treatment recommendation. However, published studies showed that some adverse events are related to the simultaneous administration of telavancin and tedizolid [42, 43]. Moreover, in our meta-analysis, the MIC values and susceptibility rates for all four antibiotics were investigated in two periods (2010–2015 and 2016–2020), and findings were very similar between the two periods. The limited use of these antibiotics and their specific action mechanisms help explain this lack of change.

In conclusion, our results demonstrated that dalbavancin, oritavancin, telavancin, and tedizolid have antibacterial activity in vitro against MRSA isolates. However, future preclinical and clinical research are necessitated to support our findings.

## Supplementary Information

**Additional file 1: **The quality assessment of included studies in this meta-analysis.

**Additional file 2: **Antibacterial activity of dalbavancin telavancin, tedizolid, and dalbavancin against MRSA isolates based on year groups.

## Data Availability

All the data in this review are included in the manuscript.

## Abbreviations

MRSA: Methicillin-resistant *Staphylococcus aureus*
MIC_50_: Minimum inhibitory concentration at which 50% of isolates were inhibited
MIC_90_: Minimum inhibitory concentration at which 90% of isolates were inhibited
S. aureus: *Staphylococcus aureus*
VRSA: Vancomycin-resistance *S. aureus*
FDA: Food and drug administration
D-Ala-D-Ala: D-alanyl-D-alanine
PRISMA: Preferred reporting items for systematic reviews and meta-analyses guidelines
MIC: Minimum inhibitory concentration
CI: Confidence interval
VRE: Vancomycin-resistant *Enterococcus*
VSE: Vancomycin-susceptible *Enterococcus*
SSTIs: Skin and soft-tissue infections
