# Supplementary material for: Antibacterial activity of recently approved antibiotics against methicillin-resistant Staphylococcus aureus (MRSA) strains: A systematic review and meta-analysis
Source: Ann Clin Microbiol Antimicrob. 2022 Aug 17;21:37. doi: 10.1186/s12941-022-00529-z (PMC9382732; doi:10.1186/s12941-022-00529-z)
Supplement: Supplementary file 1 — Additional file 1: The quality assessment of included studies in this meta-analysis. [file 12941_2022_529_MOESM1_ESM.docx]

Additional file Figure S1. Antibacterial activity of Telavancin against MRSA isolates based on year groups.

Additional file Figure S2. Antibacterial activity of tedizolid against MRSA isolates based on year groups.

Additional file Figure S3. Antibacterial activity of dalbavancin tedizolid against MRSA isolates based on year groups.
